# Temperature and electron concentration dependences of 1/*f* noise in Hg_1−*x*_Cd_*x*_Te – evidence for a mobility fluctuations mechanism

**DOI:** 10.1039/d4nr04494k

**Published:** 2025-02-21

**Authors:** Adil Rehman, Volodymyr Petriakov, Ivan Yahniuk, Aleksandr Kazakov, Iwona Rogalska, Jakub Grendysa, Michał Marchewka, Maciej Haras, Tomasz Wojtowicz, Grzegorz Cywiński, Wojciech Knap, Sergey Rumyantsev

**Affiliations:** a CENTERA Laboratories, Institute of High Pressure Physics, Polish Academy of Sciences Warsaw 01-142 Poland adilrehhman@gmail.com roumis4@gmail.com gc@unipress.waw.pl; b Terahertz Center, University of Regensburg 93040 Regensburg Germany; c International Research Centre MagTop, Institute of Physics, Polish Academy of Sciences Warsaw 02-668 Poland; d University of Rzeszów, Institute of Materials Engineering, Center for Microelectronics and Nanotechnology 35-959 Rzeszów Poland; e Centre for Advanced Materials and Technologies CEZAMAT, Warsaw University of Technology Warsaw 02-822 Poland; f Gdańsk University of Technology, Faculty of Electronics, Telecommunications and Informatics, Advanced Materials Center 80-233 Gdańsk Poland

## Abstract

Hg_1−*x*_Cd_*x*_Te is a unique material with its bandgap being tunable by temperature, pressure, and cadmium content over a wide range, from 1.6 eV to an inverted bandgap of −0.3 eV. This makes Hg_1−*x*_Cd_*x*_Te one of the key materials for infrared and terahertz detectors, whose characteristics largely depend on the material's noise properties. In this work, we investigated the low-frequency 1/*f* noise in a thick (800 nm) HgCdTe layer and in a field effect transistor (FET) with an 8 nm wide HgTe quantum well. Both structures exhibited a small contribution from contact noise and showed weak noise dependences on temperature. Investigation of the 1/*f* noise in the HgTe quantum well FET as a function of gate voltage revealed that the noise also depends weakly on electron concentration. These findings indicate that the noise properties of Hg_1−*x*_Cd_*x*_Te are similar to those of graphene, where mobility fluctuations were found to be the dominant mechanism of the 1/*f* noise.

## Abstract 2

Temperature and gate voltage dependences of the 1/*f* noise in Hg_1−*x*_Cd_*x*_Te and HgTe QW provide a strong argument for mobility fluctuations (*δμ*) as the dominant noise mechanism.

## Graphical abstract

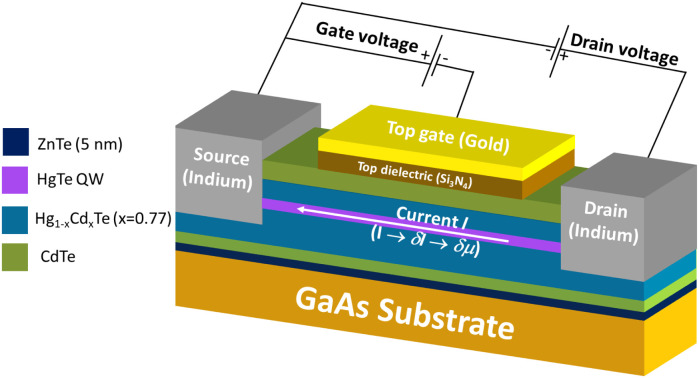

## Introduction

Mercury cadmium telluride (Hg_1−*x*_Cd_*x*_Te) has been known as a narrow-band semiconductor for over 60 years (see ref. 1 and references therein). It is one of the key materials for infrared (IR) and far-infrared (FIR) detectors and IR imaging systems.^1–4^ It also attracts significant attention for terahertz applications.^5–9^ The band structure of Hg_1−*x*_Cd_*x*_Te strongly depends on temperature (*T*), pressure, and cadmium content (*x*), which allows for tuning of the bandgap from 1.6 eV to an inverted bandgap of −0.3 eV.^1^ At a critical value of *x* = 0.12 and *T* = 300 K, the separation between the valence and conduction bands at the *Γ* point of the Brillouin zone becomes zero, and the dispersion law of electrons and light holes becomes linear. This offers great opportunities for the design of photodetectors with controlled properties and enables the exploration of interesting phenomena arising from the linear dispersion of bands, as in graphene, but in a bulk material.

The key characteristic of IR detectors is their detectivity, which is always limited by some form of noise. Thermal noise and shot noise are important for detectors operating at high modulation frequencies. For sensitive detectors operating at a low modulation frequency, the low-frequency 1/*f* and generation–recombination noise play a vital role. Therefore, many publications are devoted to studying the low-frequency noise in HgCdTe photodetectors, mainly in photodiodes.^10–17^ These studies are of great practical importance, but it is difficult to analyse the noise mechanism in devices of complex design, especially with potential barriers.

The study of low-frequency noise is also of special interest as it is a very sensitive tool used to study the properties of materials. This includes, for example, the so-called noise spectroscopy technique, which allows one to study deep levels in semiconductors not accessible by other methods.^18^ The low-frequency noise is also a very powerful tool for studying the charge transport mechanisms in disordered systems.^19,20^ To understand the nature of noise in a given material, it is important to investigate the simple ohmic structures. Studying the nature of noise in the Hg_1−*x*_Cd_*x*_Te material itself is important for understanding the 1/*f* noise mechanisms in narrow-band semiconductors and for the design of infrared and terahertz detectors, quantum computers, and other advanced applications where noise control is critical. However, there are only a few publications where the 1/*f* noise was studied in just HgCdTe materials^21–24^ or in an HgTe quantum well (QW).^25^

Here, we studied the electrical and low-frequency 1/*f* noise properties of bulk and QW Hg_1−*x*_Cd_*x*_Te structures. Our findings show that the noise properties of Hg_1−*x*_Cd_*x*_Te are unusual and resemble those of graphene. The temperature dependence of the noise in a bulk HgCdTe layer and the dependence of the noise on electron concentration in an HgTe QW provide strong arguments for mobility fluctuations as the dominant mechanism of the 1/*f* noise in Hg_1−*x*_Cd_*x*_Te. These findings contradict previous studies that attributed the noise to carrier number fluctuations in HgCdTe-based systems.^21–25^

## Results and discussion

**Fig. 1:**
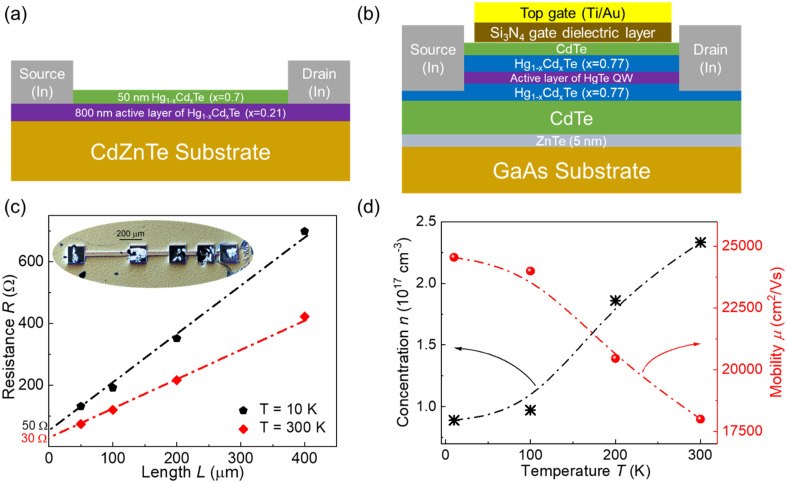
Cross-sectional schematic views of (a) the bulk HgCdTe and (b) the HgTe QW devices. (c) Resistance as a function of bulk HgCdTe device length at *T* = 10 K and *T* = 300 K. The solid symbols correspond to the individual devices of different lengths and the dash-dot lines are a guide for the eyes. The inset shows an optical image of the studied TLM structure. (d) Charge carrier concentrations and mobility of a bulk HgCdTe device at different temperatures.

In this work, two different types of Hg_1−*x*_Cd_*x*_Te-based devices were studied. The first one represents an 800 nm thick layer of Hg_0.79_Cd_0.21_Te, which we refer to as the bulk sample (see Fig. 1(a)). The second type of device was one with an HgCdTe/HgTe heterostructure. This investigated structure had a field effect transistor (FET) geometry (see the schematic view in Fig. 1(b)), which allowed tuning of carrier concentration in the HgTe QW channel by the gate voltage (*V*_G_).

### Electrical and 1/*f* noise characteristics of bulk Hg_1−-*x*_Cd_*x*_Te

The bulk HgCdTe devices are shaped as 30 μm wide strips of various lengths (see the inset in Fig. 1(c)). This allowed us to estimate the total contact resistance (2*R*_c_) and contact noise using a transmission line model (TLM) approach.^26,27^ The current–voltage (*I*–*V*) characteristics of all devices were linear within a voltage range of zero to 150 mV. Fig. 1(c) shows the resistance as a function of device length (*L*) for one of the TLM structures at two different temperatures. As seen, the dependences of resistance on the device length are linear. The intercept with the *y*-axis yields the total contact resistance. It can be seen in Fig. 1(c) that at both low and high temperatures, the total contact resistance is several times smaller than the sample resistance. The geometry of Hall bars was also included in the bulk HgCdTe structure to estimate the mobility and charge carrier concentrations. Fig. 1(d) shows the measured concentration (*n*) and mobility (*μ*) of electrons at different temperatures. It can be seen that as the temperature increases, the concentration increases and mobility decreases.

**Fig. 2:**
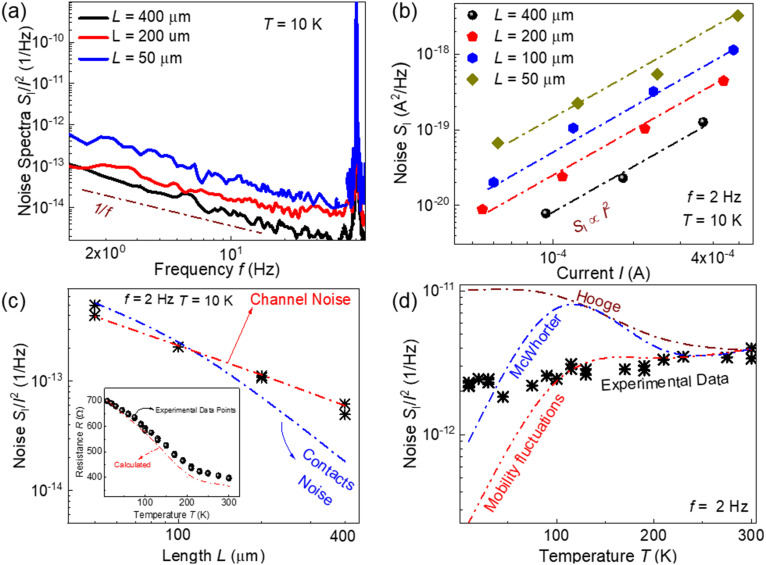
(a) Noise spectra of bulk HgCdTe devices of different lengths at 10 K. (b) Spectral noise density of current fluctuation as a function of current for different bulk HgCdTe devices. (c) Noise as a function of length for an HgCdTe TLM structure. The red and blue dash-dot lines represent the shape of the noise dependences in the case of noise from the channel and contacts, respectively. The inset shows the resistance of one of the investigated devices as a function of temperature. The red dash-dot line shows the resistance calculated using the concentration and mobility of charge carriers obtained from the Hall measurements. (d) Noise as a function of temperature for one of the investigated HgCdTe devices. The dash-dot lines show the hypothetical behavior of noise calculated assuming the Hooge formula, the McWhorter number of carriers fluctuations model, and the mobility fluctuations model.

Fig. 2(a) shows examples of the noise spectra at 10 K for several bulk HgCdTe devices of different lengths. The noise spectra of all devices have the 1/*f*^*γ*^ shape with *γ* = 1.1–1.3. Fig. 2(b) shows the spectral noise density of short circuit current fluctuations (*S*_I_) as a function of current (*I*) for the devices of different lengths at *f* = 2 Hz. It can be seen that *S*_I_ is proportional to the square of the current (*i.e. S*_I_ ∝ *I*^2^), which implies that the resistance fluctuations are responsible for the 1/*f* noise, and the current only makes these fluctuations visible.

One of the ways to distinguish between contact and channel noise is to examine the noise as a function of the device length. The spectral noise density of current fluctuations can be written as:1
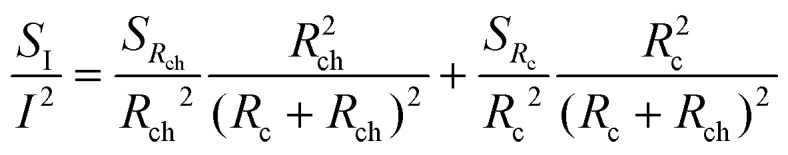


where *R*_ch_ is the channel resistance and (*S*_*R*_ch__/*R*_ch_^2^) and (*S*_*R*_c__/*R*_c_^2^) are the relative spectral noise densities of the channel and contact resistance fluctuations, respectively. If *R*_ch_ ≫ *R*_c_ and the noise from the channel dominates, the spectral noise density of current fluctuations can be written as (*S*_I_/*I*^2^) = (*S*_*R*_ch__/*R*_ch_^2^) ∝ *L*^−1^. On the other hand, if noise originates from the contacts, the spectral noise density of current fluctuations can be written as (*S*_I_/*I*^2^) = (*S*_*R*_c__/*R*_c_^2^)(*R*_c_^2^/*R*_ch_^2^) ∝ *L*^−2^.

Fig. 2(c) shows how the noise depends on the device length at 10 K. The red and blue dash-dot lines represent the shape of the dependences calculated based on the first and second terms of eqn (1) with the actual values of the contact and channel resistances. As seen, the experimental dependence of noise on the device length confirms that noise originated from the channel, not from the contacts.

The inset in Fig. 2(c) shows the resistance of one of the investigated bulk HgCdTe devices as a function of temperature. The solid symbols represent the experimental data points while the dash-dot line shows the resistance calculated using the mobility and concentration obtained from the Hall measurements. As can be seen, the resistance of the bulk HgCdTe device decreases significantly with increasing temperature and coincides well with the calculated red dash-dot line. Fig. 2(d) shows the temperature dependence of the noise in bulk HgCdTe devices. As can be seen, although the resistance changes with temperature, the noise (*S*_I_/*I*^2^) exhibits weak temperature dependence. It was also found that noise spectra do not change significantly with temperature.

We can analyse the shape of the temperature dependence of the noise based on known models. Hooge's empirical formula claims that noise is of volume origin and is inversely proportional to the total number of carriers (*N*):^28^2
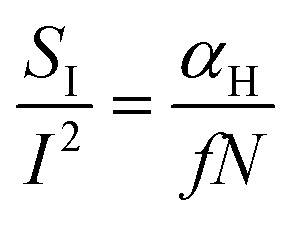
where *α*_H_ is the constant. Another option is that noise is due to the tunnelling of electrons into the adjusting Hg_0.3_Cd_0.7_Te layer or/and the CdZnTe substrate. This mechanism is described by McWhorter's model:^29^3
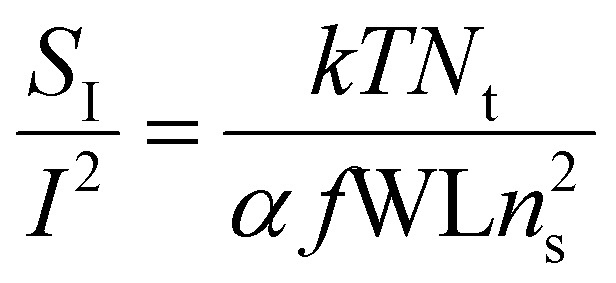
where *k* is the Boltzmann constant, *T* is the temperature, *N*_t_ is the effective trap density, WL is the channel area, *n*_s_ is the two-dimensional concentration, and *α* is the attenuation coefficient of the electron wave function within the barrier.

In general, the 1/*f* noise can also originate from the traps in the bulk. However, simple estimates show that this narrow-band semiconductor at a doping level of more than 10^17^ cm^−3^ is degenerate at all studied temperatures (*i.e.* the Fermi level is in the conduction band). This implies that all traps are filled and do not contribute to noise. Therefore, we assume that this mechanism cannot make a significant contribution to noise.

Another possible origin of the noise is mobility fluctuations. This mechanism of noise was discussed in ref. 30 and 31. In accordance with the model, this kind of noise is due to the fluctuations of the scattering cross-section (*σ*). The spectral noise density of current fluctuations for one type of scattering center can be written as:4
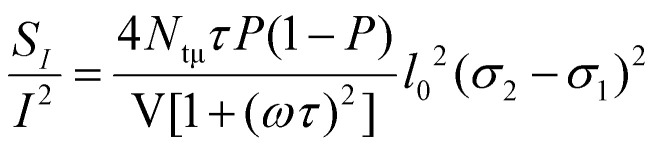


where *N*_tμ_, *τ*, *P*, *l*_0_, and *V* represent the scattering centers’ concentration, characteristic time constant, probability of scattering centers being in a state with a cross-section *σ*_1_, carrier mean free path, and volume, respectively.

It can be seen from eqn. (2) that Hooge's formula predicts that the noise (*S*_I_/*I*^2^) should depend on temperature as 1/*N*. McWhorter's model predicts that the noise depends on temperature as ∝*T*/*n*_s_^2^ for uniform energy distribution of traps. To obtain the 1/*f* noise due to the mobility fluctuations, one needs to integrate eqn (4) over multiple scattering centers with a wide distribution of characteristic times (*τ*). Although the result depends on the actual distribution of the scattering centers, as a first approximation, we can assume that noise linearly increases with increasing temperature (see ref. 31 for the detailed analysis). Since mobility is proportional to the mean free path, we can write the spectral noise density of current fluctuations in the case of the mobility fluctuations mechanism as:5
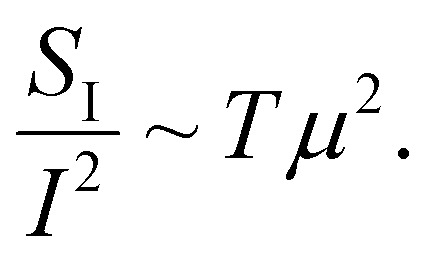


The red, blue, and brown dash-dot lines in Fig. 2(d) were calculated based on the three models discussed above. The noise values were fitted to the experimental data at *T* = 300 K.

In eqn (2), the Hooge parameter is taken *α*_H_ = 8 × 10^−3^. This value is approximately an order of magnitude smaller than those reported in ref. 10 and 24, larger than the values presented in ref. 22 for the HgCdTe system, and comparable to the value reported for graphene.^32^ Although eqn (2) represents just an empirical formula, it is often used to characterize the noise level. Therefore, we can state that our samples exhibit an average noise level. It is important to note also that the noise level depends on many factors including composition, type and level of doping, temperature, and sample geometry.

In general, the McWhorter model, represented by eqn (3), allows one to extract the trap density. However, it was derived for two-dimensional conducting channels of FETs and contains the two-dimensional concentration. Since we are dealing with relatively thick samples, we cannot define this concentration and can only make judgements about the shape of the dependence predicted using eqn (3). A similar argument can be used for eqn (4) because it also contains several unknown parameters.

Nevertheless, it can be seen that the mobility fluctuations model fits the experimental data very well at high temperatures. At low temperatures, none of the models are consistent with the experimental data. However, it is important to mention that this is a more general problem. The majority of the models predict that the noise decreases and tends to zero with the temperature approaching zero (one of the exceptions is Hooge's formula). However, this is rarely observed experimentally in any electronic system. In graphene, for example, the 1/*f* noise either weakly depends on temperature^32^ or sharply increases below 10 K.^33^ The reason for this kind of noise behavior at cryogenic temperatures is not clear yet.

Therefore, based on the temperature dependence of noise at *T* > 100 K, we conclude that the mobility fluctuations model is the most realistic mechanism of the 1/*f* noise in these Hg_1−*x*_Cd_*x*_Te samples.

### Electrical and 1/*f* noise characteristics of the HgTe QW

**Fig. 3:**
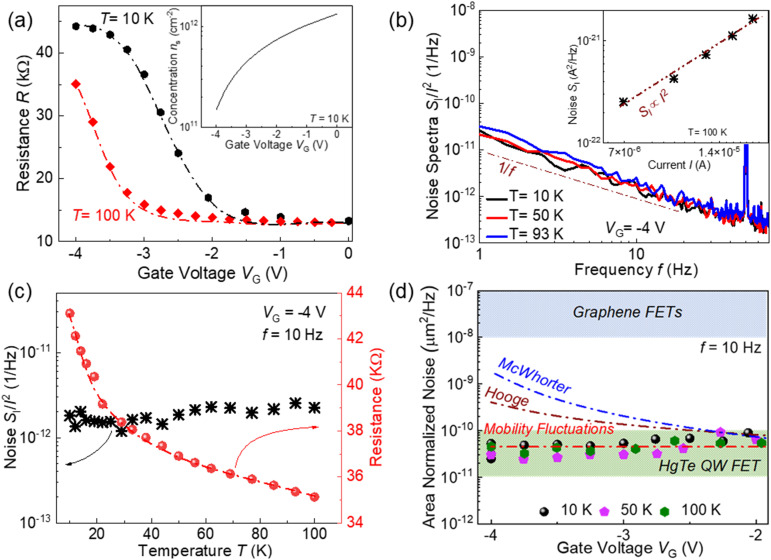
(a) Resistance of the HgTe QW-based FET as a function of gate voltages at two temperatures. The inset shows the gate voltage dependence of the electron concentration in the QW at 10 K. (b) Noise spectra at different temperatures for the HgTe QW-based FET. The inset shows the spectral noise density of current fluctuations as a function of current at 100 K. (c) Resistance and noise as a function of temperature at *V*_G_ = −4 V. (d) Symbols showing the area-normalized noise as a function of gate voltage at three different temperatures. The upper dashed area corresponds to the noise level in graphene devices.^37^ The red, blue, and brown dash-dot lines show the hypothetical behavior of the gate voltage dependences of noise assuming the Hooge formula, the McWhorter number of carriers fluctuations model, and the mobility fluctuations model.

We also investigated the electrical and low-frequency noise characteristics of the HgTe QW structures. Fig. 3(a) shows the dependence of the HgTe QW-based device resistance on the gate voltage at two temperatures. It can be seen that the device behaves as an n-channel FET. For gate voltages *V*_G_ < −1.5 V, the resistance increases, manifesting a decrease in the electron concentration, while for gate voltages *V*_G_ > −1.5 V, the resistance only weakly depends on gate voltage and temperature, indicating the dominance of the contact resistance. The inset in Fig. 3(a) shows the gate voltage dependence of the electron concentration in the QW calculated as *n*_s_ = *C*(*V*_G_ − *V*_t_)/*q*, where *C* is the gate capacitance per unit area, *q* is the elementary charge, and *V*_t_ is the threshold voltage taken to be *V*_G_ = −4.5 V.

The dependence of spectral noise density on short circuit current fluctuations (at *f* = 10 Hz) as a function of current at *V*_G_ = −4 V and *T* = 100 K is shown in the inset in Fig. 3(b). It can be seen that *S*_I_ is proportional to the square of the current. Since channel resistance dominates at *V*_G_ = −4 V (see Fig. 3(a)), this implies, as described earlier, that channel resistance fluctuations are responsible for the 1/*f* noise origin. The noise spectra of the studied device at different temperatures are shown in Fig. 3(b). It can be seen that the noise spectra have the 1/*f* shape, and, as with the bulk HgCdTe device, neither the noise amplitude nor the shape of the spectrum changes significantly with temperature.

Fig. 3(c) shows the resistance and noise of the HgTe QW-based FET as a function of temperature at *V*_G_ = −4 V, where the device resistance is dominated by the channel resistance. The bandgap of the HgTe QW strongly depends on temperature.^34,35^ The detailed band structure calculations based on the eight-band *k*·*p* Hamiltonian for (013)-oriented heterostructures, which directly takes into account the interactions between *Γ*_6_, *Γ*_8_, and *Γ*_7_ bands in bulk materials, were performed in ref. 36. The calculations show that at *T* = 10 K the band structure is inverted, and with increasing temperature, the band gap decreases and becomes zero at *T* ≈ 100 K. It can be seen that resistance decreases as temperature increases, but the noise, despite the significant temperature dependence of the band gap,^36^ shows only a weak temperature dependence. This behaviour is similar to that observed for a bulk HgCdTe device (see Fig. 2(d)).

It can also be seen in Fig. 3(a) that the slopes of the resistance *versus* temperature dependences are virtually the same for *T* = 10 K and *T* = 100 K. These slopes correspond to the transconductances, which are determined by the mobility. The similarity in slopes means that the mobilities remain the same at these temperatures. Therefore, despite the temperature dependence of the bandgap, the change in the mobility is small for this temperature range, and in accordance with eqn (4), noise only weakly depends on temperature, which is consistent with the experiment.

Fig. 3(d) shows the area-normalized noise (Area × *S*_I_/*I*^2^) of the studied HgTe QW-based FET (at *f* = 10 Hz) as a function of *V*_G_ at three different temperatures. The noise (*S*_I_/*I*^2^) was normalized to the sample area to facilitate comparison with graphene devices (light blue area in Fig. 3(d)). The typical noise range for graphene devices is in the range of 10^−8^–10^−7^ μm^2^ Hz^−1^.^37^ It is seen that the noise level in the studied HgTe QW is smaller than in graphene and does not depend on *V*_G_.

The dash-dot lines in Fig. 3(d) show the shapes of the gate voltage dependences of noise assuming Hooge's formula, McWhorter's number of carriers fluctuations, and mobility fluctuations models assuming mobility is independent of gate voltage (concentration). It is important to emphasize that, in contrast to the number of carriers fluctuations mechanism of noise, the contribution to noise from the mobility fluctuations does not depend on either the total number of carriers or their concentration. Therefore, only the mobility fluctuations mechanism is consistent with the observed weak dependence of noise on the gate voltage.

As shown above, the temperature dependence of noise, both in the bulk and QW samples, also complies with the mobility fluctuations mechanism. These findings contradict previous studies that attributed the noise to carrier number fluctuations in HgCdTe-based systems.^21–25^ HgCdTe is a complex materials system, with its properties strongly influenced by factors such as composition, doping, geometry, and defect concentration. Therefore, it is quite possible that the noise mechanism depends on these parameters as well. However, we can state that the mobility fluctuations mechanism in Hg_1−*x*_Cd_*x*_Te is possible under certain conditions.

This means that the noise properties of Hg_1−*x*_Cd_*x*_Te can be similar to those of graphene, where the mobility fluctuations mechanism is considered as the dominant source of the 1/*f* noise.^38,39^

## Conclusions

The low-frequency 1/*f* noise in the bulk HgCdTe structure and the HgTe QW was investigated over a wide temperature range. Both structures exhibited weak temperature dependence on the noise. A study of the 1/*f* noise in FETs based on the HgTe QW showed that the noise was also weakly dependent on electron concentration. These results indicate the similarity of the noise properties of Hg_1−*x*_Cd_*x*_Te and graphene, provide strong arguments for the mobility fluctuations mechanism of the 1/*f* noise in Hg_1−*x*_Cd_*x*_Te, and open pathways to optimize the performance of HgCdTe devices for infrared and terahertz detection applications.

## Experimental methods

### Bulk HgCdTe growth and device fabrication

The bulk Hg_1−*x*_Cd_*x*_Te structure was grown on a polished CdZnTe (211) substrate by molecular beam epitaxy (MBE) in a Riber Compact 21 MBE system equipped with a liquid Hg source and reflection high-energy electron diffraction (RHEED) system. The growth rate and composition (*x* = 0.21) of the Hg_1−*x*_Cd_*x*_Te epilayers were controlled and the process was monitored in a real time using the *in situ* RHEED system (see ref. 40 for the detailed process). The grown stack consisted of a 50 nm Hg_0.3_Cd_0.7_Te cap layer and an active 800 nm thick Hg_0.79_Cd_0.21_Te layer on the CdZnTe substrate (see Fig. 1(a)). To fabricate ohmic contacts, the windows in the cap layer were first opened using standard photolithography and wet etching in a 0.05% bromine–methanol solution. Afterwards, indium (In) contacts were deposited using the electrodeposition technique. The final device configurations were obtained in a second photolithography step and wet mesa etching to identify and separate each device.

### HgCdTe/HgTe heterostructure growth and device fabrication

The HgCdTe/HgTe heterostructures were grown by MBE on a (013)-oriented GaAs substrate.^4^ The 8 nm QW was encapsulated in HgCdTe barrier layers with high molar Cd content (see Fig. 1(b) for the grown stack details). Electron beam lithography and wet etching in 0.08% bromine–methanol solution were used to define 5 × 3 μm^2^ devices, and indium contacts, acting as source and drain terminals, were made. The plasma-enhanced chemical vapor deposition technique was employed to deposit a 140 nm thick Si_3_N_4_ layer as a top gate dielectric. Titanium/gold (Ti/Au) metal layers (10/110 nm) acting as top gate contacts were thermally evaporated. A detailed description of the device fabrication process can be found elsewhere.^41^

### Electrical and noise measurements

All measurements were conducted by placing the devices on the sample stage of a closed-cycle cryogenic probe station (Lake Shore Inc., CRX-VF),^42^ and special probes were used for the electrical and low-frequency noise measurements as a function of temperature without the need for probe lifting. Electrical measurements were performed by using a semiconductor parametric analyzer (Keithley 4200A-SCS). For the noise studies, the voltage fluctuations across the load resistor (*R*_L_) were amplified by a low-frequency noise amplifier and analyzed using a fast Fourier transform “PHOTON” dynamic signal analyzer. The obtained spectral noise density (*S*_v_) was then converted into the spectral noise density of short circuit current fluctuations (*S*_I_) as *S*_I_ = *S*_v_[(*R*_L_ + *R*_D_)/(*R*_L_*R*_D_)]^2^. Here, *R*_D_ is the device resistance. The background noise of the system was always 15–20 dB smaller than that of the studied devices and was checked by replacing the device with a metal resistor of the same value as *R*_D_. To obtain the precise value of noise at a given frequency, the noise spectra were fitted to a straight line in the form of 1/*f*^*γ*^. This procedure increased the accuracy of the noise value reading, especially at low frequencies.

## Author contributions

S. R. conceived the idea of this study. A. R. performed the measurements and analyzed the data. I. Y., V. P., M. H., and A. K. fabricated the devices. I. R., J. G., and M. M. grew the HgCdTe wafer. W. K., G. C., and T. W. contributed to the data analysis. S. R. and A. R. wrote the manuscript. All authors participated in the manuscript preparation.

## Data availability

The data supporting the findings of this study are available within the article. Additional data that support the finding are available from the corresponding author upon reasonable request. The raw data sets are available from RepOD at https://repod.icm.edu.pl.

## Conflicts of interest

There are no conflicts to declare.
